# An observational cohort study evaluating PrEP reach, engagement and persistence through a community‐based mobile clinic in Miami‐Dade County, Florida

**DOI:** 10.1002/jia2.26362

**Published:** 2024-10-14

**Authors:** Susanne Doblecki‐Lewis, Ariana Johnson, Katherine Klose, Katherine King, Gilianne Narcisse, Stefani Butts, Patrick Whiteside, Erin Kobetz, Mario Stevenson

**Affiliations:** ^1^ Division of Infectious Diseases Department of Medicine University of Miami Miller School of Medicine Miami Florida USA; ^2^ Division of Prevention Science Department of Medicine University of Miami Miller School of Medicine Miami Florida USA; ^3^ Prevention305 Miami Beach Florida USA; ^4^ Division of Medical Oncology Department of Medicine University of Miami Miller School of Medicine Miami Florida USA

**Keywords:** PrEP, HIV prevention, men who have sex with men, retention, community, mobile clinics

## Abstract

**Introduction:**

Barriers to pre‐exposure prophylaxis (PrEP) access have limited its reach to priority populations. Community‐based mobile clinics have potential to broaden PrEP engagement. We evaluated reach and persistence for fixed and mobile clinic cohorts in Miami‐Dade County, Florida.

**Methods:**

This observational cohort study analysed data from 1896 clients engaged through our fixed or mobile clinic from August 2018 to March 2023. Services were offered at no cost to clients. The same staff and package of barrier‐lowering strategies was deployed across fixed and mobile clinic sites. Chi‐square and Fisher's exact test or the Kruskal–Wallis test were used to test for differences in characteristics across sites as well as across services sought. Kaplan–Meier curves were generated to evaluate persistence on PrEP and in care, defined as completion of at least one clinic visit (including PrEP prescribing, for PrEP persistence, or for any reason, for persistence in care) within 24 weeks of the prior visit. Cox proportional hazards models were used to evaluate risk factors for discontinuation of PrEP or clinic care by 48 weeks by gender, race, ethnicity, insurance status and site.

**Results:**

The fixed and mobile clinics reached 781 and 1109 clients, respectively, during the study period. The median client age was 35 years; the majority (70.4%) of clients were cisgender men, identified as Hispanic/Latino (62.5%) and were men who have sex with men (54.5%). The mobile clinic extended reach to a higher proportion of cisgender women (32.1% mobile vs. 12.9% for fixed clinic), Black clients (34.5% vs. 13.1%) and older clients (median 37 vs. 33 years) compared with the fixed setting. Uninsured individuals, men and those who initiated services in the mobile clinic were more likely to continue PrEP to 48 weeks (HR: 1.20, *p* = 0.01; HR: 2.02, *p*<0.01; HR: 1.68, *p*<0.01, respectively). Persistence did not differ by race or ethnicity.

**Conclusions:**

A mobile clinic strategy for PrEP engagement can increase reach to key populations underrepresented in HIV prevention care including cisgender women and Black clients. Persistence in PrEP was increased for the mobile clinic cohort, suggesting an additional benefit to this modality beyond other barrier‐lowering strategies employed in our fixed and mobile clinics.

## 1 INTRODUCTION

Pre‐exposure prophylaxis (PrEP) for the prevention of HIV presents a significant opportunity to reduce HIV incidence and is a critical pillar of the US Ending the HIV Epidemic plan [1]. However, the public health impact of PrEP will depend on its utilization among key populations [2].

Miami‐Dade County (Miami), in South Florida, an area of the United States where rates of newly diagnosed people living with HIV (PLWH) are particularly high [2, 3], PrEP use has not scaled proportionately to need [4], and many potential PrEP candidates may have barriers to obtaining PrEP [5, 6]. Disparities in PrEP initiation by socio‐economic status, educational level, race and ethnicity have been described nationally [7, 8]. PrEP access in Miami may be especially limited, as individuals in this region are more likely to be economically disadvantaged, have challenges related to immigration status and to be un/under‐insured than those residing elsewhere in the United States [9, 10]. Other social and structural factors, such as stigma, transportation barriers, inconvenient hours of operation and difficulties finding a willing PrEP provider, exacerbate disparities in PrEP access, particularly for minorities including Latino and Black men who have sex with men (MSM) and Black women [6, 9, 11–13]. We have previously described PrEP provider access and transportation issues as significant barriers to PrEP initiation in Miami [6, 14].

Mobile HIV testing programmes have demonstrated success in overcoming stigma and transportation‐related barriers and increasing HIV testing rates within high‐incidence areas globally, allowing reach to populations that are not well represented in traditional clinic settings [15, 16, 17, 18, 19, 20]. Mobile strategies have also been frequently deployed as part of HIV treatment programmes in sub‐Saharan Africa, particularly in rural regions [21, 22, 23, 24]. The use of mobile clinics for PrEP was determined to be feasible and acceptable as part of a sexual healthcare programme for adolescent girls and young women in South Africa [25] and is currently under evaluation as part of comprehensive care for people who use drugs at multiple locations in the United States [26, 27]. To our knowledge, long‐term data from a mobile clinic model for PrEP in the United States has not previously been available.

In this manuscript, we describe demographics, utilization and persistence in services initiated through a mobile clinic providing HIV and sexually transmitted infection (STI) testing and treatment as well as PrEP care (including provider evaluation, laboratory evaluation, PrEP prescription, counselling, condoms and lubricant, and PrEP navigation) over more than 4 years of implementation, and compare this delivery strategy with that of our fixed clinic.

## 2 METHODS

This observational study evaluated two cohorts of individuals (*n* = 1896) initiating services other than HIV care at (1) the University of Miami Mobile PrEP Clinic; or (2) the Rapid Access Wellness fixed clinic from August 2018 to March 2023.

### 2.1 Mobile clinic setting

**Figure 1:**
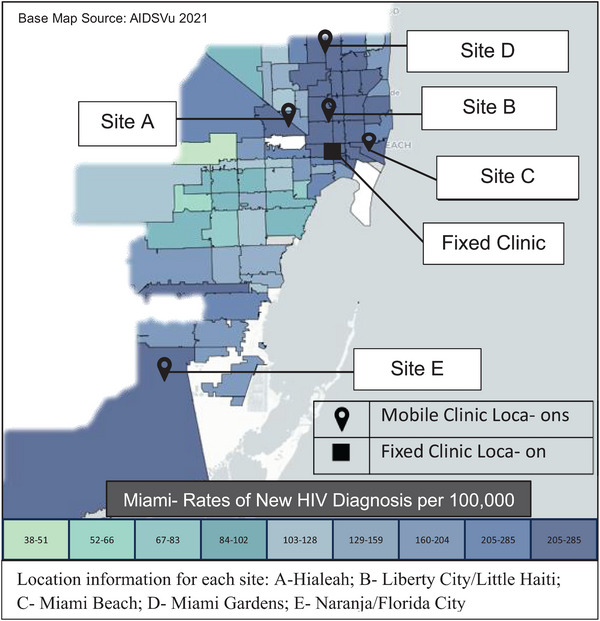
Fixed and mobile clinic locations within Miami‐Dade County. Base map displays rates of new diagnoses by zip code 2021 (base map source: AIDSVu) [20].

Established in 2018, the University of Miami Mobile PrEP Clinic is a customized provider‐staffed mobile clinic that provides low‐barrier PrEP, post‐exposure prophylaxis (PEP) and STI care. Services are provided at locations chosen based on new HIV acquisition rates and community needs for PrEP services (Figure 1). Locations are reviewed quarterly with the Florida Department of Health and adjusted based on epidemiological data. Since project initiation, two sites remain unchanged, two moved within the same county region and one new site was added in November 2019. Site placement involves community engagement and preparation before mobile clinic deployment and ongoing activities thereafter as described elsewhere [28]. The mobile clinic, staffed by a medical provider (advanced practice nurse or physician) and HIV/PrEP counsellor/navigator, rotates through sites weekly or biweekly. Clients are directed to the mobile clinic through partner organization referral, neighbourhood outreach and social media. The schedule is published on social media and shared with community partners. Outreach activities include open houses, events, a monthly “lunch and learn” series, radio and door‐to‐door distribution of programme materials. Due to COVID‐19, mobile services were suspended from March to September 2020. Services continued through telehealth and in‐person visits at the fixed clinic.

### 2.2 Fixed clinic setting

In March 2020, a fixed clinic was launched, serving as a base for appointments, telehealth, assistance with prescriptions and service access, and laboratory functions. The fixed clinic is open daily including evenings. All services offered through the fixed clinic, except for telehealth, are also offered in the mobile setting.

### 2.3 Overall description of programme services and strategies for mobile and fixed clinics

Services at fixed and mobile clinics are free for clients regardless of insurance. Staff are funded by contracts with the Florida Department of Health and income is generated through federal pharmacy rebate programmes. Medications are provided through electronic prescription to partner pharmacies, funded by insurance or available programmes for uninsured or underinsured clients. Navigators assist with prescriptions, enrolment in assistance programmes and adherence counselling. Laboratory testing and supplies are funded by the Florida Department of Health, the City of Miami Beach and in‐kind agreements with the Miami Health Department HIV/STI Program.

Staff, who are shared between the fixed and mobile clinics, are multilingual (English, Spanish, Haitian Creole), and representative of the communities served. Training and education provided to staff through our community partners has included healthcare considerations for sexual and gender minority clients, trauma‐informed care, neighbourhood history, the influence of structural racism on healthcare and neighbourhood walking tours with community leaders.

Services, including laboratory screening, provider evaluation, counselling, condoms and lubricant, are identical at the fixed and mobile sites. Mobile clinic clients may elect to receive follow‐up in the mobile or fixed clinic. Transportation to the fixed clinic is provided at no cost. Client navigators facilitate access programmes such as prescription payment assistance, prior authorizations, medication adherence and PEP when appropriate. On‐site treatment for gonorrhoea, chlamydia and syphilis is offered at all sites since July 2019. Rapid entry into HIV care with immediate initiation of antiretrovirals for those with a reactive HIV test result has been offered onsite since April 2021. HIV treatment visit data are not included in this manuscript.

Client‐related data, including demographics, substance use, insurance status, sexual behaviours and number of sexual partners, are recorded in a REDCap database. Non‐rapid test results are transmitted to clients through the electronic medical record patient portal and/or by telephone and recorded in the REDCap database. Pharmacy pick‐up data were available for a subset of clients initiating PrEP between March 2020 and March 2023. Miami‐Dade County demographic data and HIV incidence numbers were collected from open‐access sources, including the 2022 census data and 2021 AIDSVu data [29, 30].

The University of Miami Institutional Review Board reviewed the data collection and analysis plan and determined that this demonstration project did not qualify as human subject research.

### 2.4 Analysis

Demographic characteristics, sexual behaviour, HIV risk perception, reported substance use and STI testing results, and PrEP use are presented using descriptive statistics. Baseline and follow‐up characteristics for the study cohorts were described with means and standard deviations for continuous variables and proportions for categorical variables. We used the chi‐square and Fisher's exact tests for categorical variables and the Kruskal–Wallis test for continuous variables to test for differences in characteristics across PrEP initiation sites (first comparing those who initiated services on a fixed clinic or mobile clinic, then comparing across all mobile clinic locations) as well as across services sought (comparing those who came for PrEP to those who came for other services) (Tables 1 and 2). Other services include HIV testing, STI testing, STI treatment and PEP. Additionally, we gathered demographic information for newly diagnosed PLWH in Miami‐Dade County in 2021 as well as overall county demographics to compare to the clients served in our mobile and fixed clinics (Table 3).

**Table 1:** Baseline characteristics of clients in fixed and mobile clinic cohorts

	Fixed and mobile clinics (*N* = 1890)	Mobile (*N* = 1109)	Fixed (*N* = 781)	*p*‐value^*^	Other services^a^ (*N* = 825)	PrEP services (*N* = 1065)	*p*‐value^**^
**Clinic type**							<0.01
Fixed	781 (41.3%)	N/A	N/A	N/A	223 (27.1%)	558 (52.4%)	<0.01
Mobile	1109 (58.7%)	N/A	N/A	N/A	602 (72.9%)	507 (47.6%)	<0.01
**Services initiated**				<0.01			
PrEP services	1065 (56.3%)	507 (45.7%)	558 (71.4%)	<0.01	N/A	N/A	N/A
Other services	825 (43.7%)	602 (54.3%)	223 (28.6%)	<0.01	N/A	N/A	N/A
**Median age**	35.0	37.0	33.0	< 0.01	36.0	34.0	< 0.01
**[Min, Max]**	[17, 81]	[19, 81]	[17, 77]		[17, 81]	[19, 81]	
**Gender**				< 0.01			< 0.01
Cisgender Man	1337 (70.7%)	727 (65.5%)	610 (78.2%)	< 0.01	420 (50.7%)	917 (86.1%)	<0.01
Cisgender Woman	457 (24.2%)	356 (32.1%)	101 (12.9%)	< 0.01	368 (44.6%)	89 (8.3%)	<0.01
Transgender/ Gender non‐conforming	96 (5.1%)	26 (2.4%)	70 (8.9%)	0.05	37 (4.7%)	59 (5.6%)	0.05
**Race**				< 0.01			< 0.01
White	1078 (57.3%)	576 (51.9%)	502 (64.3%)	0.03	351(42.7%)	727 (68.3%)	< 0.01
Black	484 (25.6%)	382 (34.5%)	102 (13.1%)	< 0.01	341 (41.3%)	143 (13.4%)	< 0.01
More than one Race/other	266 (13.9%)	103 (9.3%)	163 (20.8%)	0.02	86 (10.3%)	180 (16.9%)	< 0.01
Asian	62 (3.2%)	48 (4.3%)	14 (1.8%)	< 0.01	47 (5.7%)	15 (1.4%)	< 0.01
**Identifies as Hispanic/Latino**	1181 (62.5%)	618 (55.7%)	563 (72.1%)	< 0.01	384 (46.7%)	797 (74.9%)	< 0.01
**Born in the United States**	823 (43.5%)	566 (51.0%)	257 (32.9%)	< 0.01	498 (60.1%)	325 (30.5%)	< 0.01
**Baseline HIV test reactive**	48 (2.5%)	11 (1.0%)	37 (4.7%)	0.05	22 (2.7%)	26 (2.4%)	0.51
**Acute bacterial STI at initial visit**	361 (19.1%)	172 (15.5%)	189 (24.2%)	0.06	94 (11.3%)	267 (25.2%)	<0.01
**Median # sex partners (past 12 months) [Min, Max]**	3.50 [0,300]	3.00 [0, 300]	4.00 [0, 200]	< 0.01	2.00 [0, 200]	5.00 [0, 300]	0.05
**Identifies as MSM**	1030 (54.5%)	484 (43.6%)	546 (69.9%)	< 0.01	151 (18.3%)	879 (82.5%)	<0.01
**Drug use (past 12 months)**							
Hallucinogens	176 (9.3%)	91 (8.2%)	85 (10.9%)	0.06	38 (4.6%)	138 (12.9%)	0.01
Stimulants	184 (9.7%)	102 (9.2%)	82 (10.5%)	0.39	42 (5.1%)	142 (13.3%)	<0.01
Opioids	36 (1.9%)	27 (2.4%)	9 (1.2%)	0.07	10 (1.2%)	26 (2.4%)	0.08
**Insured at baseline visit** ^b^ **(*n* = 998)**	458 (43.0%)	243 (48.0%)	297 (53.3%)	0.04	N/A	458 (43.0%)	N/A

^a^ Other services included HIV and STI testing, STI treatment and PEP.

^b^ Insurance information was only available for those who came for PrEP services.

* *p*‐values reflect chi‐square or *t*‐tests comparing clinic type (mobile vs. fixed).

** *p*‐values reflect chi‐square or *t*‐tests comparing service type (PrEP services vs. other services).

**Table 2:** Client demographics by mobile clinic location

	Site A (*N* = 155)	Site B (*N* = 268)	Site C (*N* = 331)	Site D (*N* = 106)	Site E (*N* = 139)	Overall (*N* = 999)	*p*‐value
Median age	36.0	39.0	35.0	32.0	42.5	37.0	< 0.01
[Min, Max]	[21.0, 69.0]	[21.0, 79.0]	[19.0, 81.0]	[22.0, 70.0]	[25.0, 76.0]	[19.0, 81.0]	
**Gender**							< 0.01
Cisgender man	112 (72.3%)	167 (62.3%)	291 (87.9%)	40 (37.7%)	61 (43.9%)	671 (67.2%)	< 0.01
Cisgender woman	41 (26.5%)	94 (35.1%)	32 (9.7%)	64 (60.4%)	76 (54.7%)	307 (30.7%)	< 0.01
Transgender/ gender non‐conforming	2 (1.3%)	7 (2.6%)	8 (2.4%)	2 (1.9%)	2 (1.4%)	21 (2.1%)	< 0.01
**Race**							< 0.01
White	127 (81.9%)	79 (29.5%)	244 (73.7%)	8 (7.5%)	65 (46.8%)	523 (52.4%)	< 0.01
Black/African American	7 (4.5%)	169 (63.1%)	32 (9.7%)	68 (64.2%)	57 (41.0%)	333 (33.3%)	< 0.01
More than one race/other	17 (7.1%)	20 (4.5%)	48 (14.5%)	12 (11.3%)	8 (5.8%)	97 (9.7%)	< 0.01
Asian	4 (6.5%)	8 (2.9%)	7 (2.1%)	18 (17.0%)	9 (6.4%)	46 (4.6%)	< 0.01
**Identifies as Hispanic/Latino**	136 (87.7%)	96 (35.8%)	226 (68.3%)	34 (32.1%)	76 (54.7%)	568 (56.9%)	< 0.01
**US born**	40 (25.8%)	173 (64.6%)	113 (34.1%)	83 (78.3%)	92 (66.2%)	501 (50.2%)	< 0.01
**Insured at baseline**	31 (15.6%)	44 (22.1%)	107 (53.8%)	7 (3.5%)	10 (5.0%)	199 (43.0%)	0.05

Location information for each site: A‐Hialeah; B‐Liberty City/Little Haiti; C‐Miami Beach; D‐Miami Gardens; E‐Naranja/Florida City.

**Table 3:** PrEP cohorts and county demographics (new HIV diagnoses and overall county demographics)

	UM PrEP cohorts			Miami‐Dade County	
	Mobile cohort (*N* = 1109)	Fixed cohort (*N* = 781)	Combined mobile and fixed cohorts (*N* = 1890)	New HIV diagnoses (*N* = 814)	Overall county demographics (*N* = 2,758,636)
**Age**					
Median [Min, Max]	37.0 [19, 81]	33.0 [19, 77]	35.0 [19, 81]	39.0 [13, 75]	41.1 [0, 106]
**Sexual or gender identity**					
Male or man^*^	727 (65.5%)	610 (78.2%)	1337 (70.7%)	689 (84.7%)	1,341,202 (48.6%)
Female or woman^*^	356 (32.1%)	101 (12.9%)	457 (24.2%)	120 (14.7%)	1,417,434 (51.4%)
Transgender/gender non‐conforming^*^	26 (2.4%)	70 (8.9%)	96 (5.1%)	5 (0.6%)	
**Race**					
White^*^	576 (51.9%)	502 (64.3%)	1078 (57.3%)	604 (74.2%)	2,095,573 (75.9%)
Black/African American	382 (34.5%)	102 (13.1%)	484 (25.6%)	202 (24.8%)	450,402 (16.4%)
More than one race/other^*^	103 (9.3%)	163 (20.8%)	266 (13.8%)	7 (0.9%)	171,178 (6.2%)
Asian	48 (4.3%)	14 (1.8%)	62 (3.3%)	3 (0.4%)	41,483 (1.5%)
**Born in the United States**	566 (51.0%)	257 (32.9%)	823 (43.4%)	314 (38.6%)	1,489,663 (54.0%)
**Identifies as Hispanic/Latino**	618 (55.7%)	563 (72.1%)	1181 (62.5%)	519 (66.5%)	1,974,449 (71.6%)

*Note*: A Z‐test of proportions was used to compare the overall mobile and fixed clinics to the Miami‐Dade County new HIV diagnosis.

* Indicates a *p*‐value of less than 0.05.

Of those who came for PrEP services (*n* = 1065) and those who were eligible for their first PrEP follow‐up (> 12 weeks since PrEP initiation), we assessed persistence in care through attendance at appointments (mobile clinic, fixed clinic or telehealth). PrEP persistence is defined as having completed at least one clinic visit that resulted in the writing of a PrEP prescription within 24 weeks of the prior visit. Persistence in care is defined as having attended at least one clinic visit for any reason within 24 weeks of the prior visit, regardless of whether PrEP was addressed. Pharmacy prescription fill rate was available for a subset of clients initiating PrEP through designated pharmacies able to provide these records.

**Figure 2:**
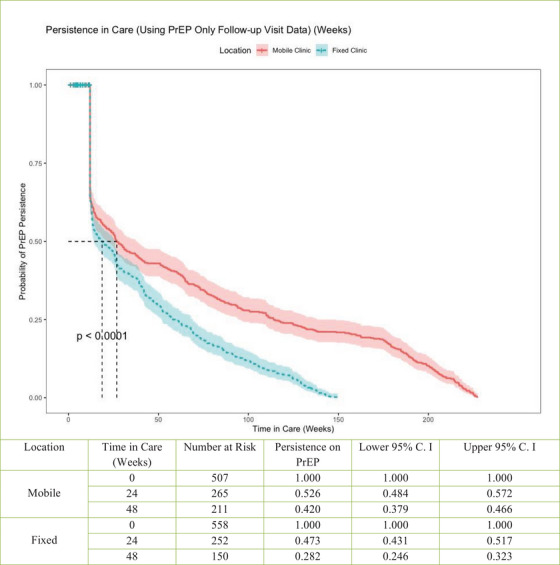
Persistence on PrEP with time in care including only visits that resulted in a PrEP prescription by initiation site (fixed or mobile clinic). Includes those in care from 9/24/2018 to 3/31/2023, excluding those initiating PrEP from March to September 2020.

**Figure 3:**
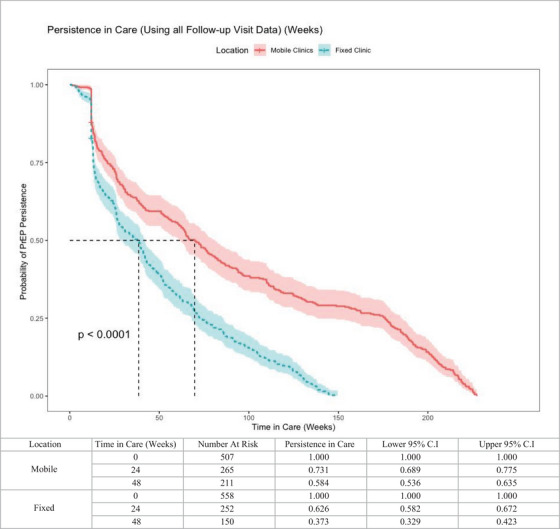
Persistence in care after initiation of PrEP, including all PrEP, HIV testing and STI testing/treatment appointments, by initiation site (fixed or mobile clinic). Includes those in care from 9/24/2018 to 3/31/2023, excluding those initiating PrEP from March to September 2020.

**Figure 4:**
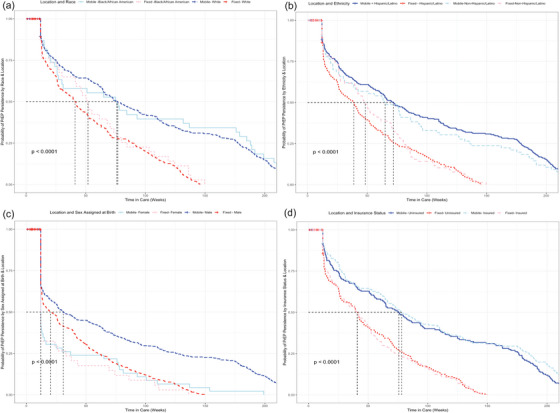
(A) Persistence on PrEP by race (Black/African American or White) and clinic cohort; (B) persistence on PrEP by ethnicity (Hispanic/Latino or non‐Hispanic/Latino and clinic cohort; (C) persistence on PrEP by sex assigned at birth (male or female) and clinic cohort; (D) persistence on PrEP by insurance status (uninsured or insured) and clinic cohort.

Outcomes of interest were persistence in PrEP care to 24 and 48 weeks and the difference in persistence in PrEP care between our mobile locations and fixed site. Kaplan–Meier curves were generated, and the log‐rank test was used to compare longitudinal persistence on PrEP (Figure 2) or in clinic care (Figure 3) and the location of PrEP initiation (mobile vs. fixed). The Kaplan–Meier curves used discontinuation from clinic care (no subsequent clinic visits for 24 weeks) as the event of interest for persistence in clinic care, and discontinuation of PrEP care (no subsequent PrEP prescription visit for 24 weeks) as the event of interest for persistence on PrEP. For this analysis, follow‐up began at enrolment and ended at the time of PrEP discontinuation or last visit before 1 March 2023, whichever occurred first. We calculated the cumulative time in care as the sum of total time between appointments in which a PrEP prescription was initiated or continued, with follow‐up appointment occurring within 24 weeks of previously scheduled appointment. Kaplan−Meier curves were also generated along with log‐rank tests to compare persistence on PrEP or in clinic care and the location of PrEP initiation in addition to race, ethnicity, sex assigned at birth and insurance status (Figure 4). Analyses using insurance status included a subset of data matched to the pharmacy fill data (*n* = 998).

Subgroup analysis was performed on individuals who were matched to the pharmacy prescription fill data and initiated PrEP prior to March 2022 (initiated care at least 1 year prior to March 2023; *n* = 919). Cox proportional hazard models were used to estimate adjusted Hazard Ratio (aHR) of risk factors for discontinuation of care by 48 weeks adjusting for sex assigned at birth, race, ethnicity, insurance status and location of visit (Table 4).

**Table 4:** Adjusted persistence on PrEP and in care to 48 weeks (*N* = 919)

	PrEP persistence^a^				Persistence in care^b^			
	Adjusted hazard ratio (aHR)	Lower C.I.	Upper C.I.	*p*‐value	Adjusted hazard ratio (aHR)	Lower C.I.	Upper C.I.	*p*‐value
**Mobile** versus **fixed location**	**1.68**	**1.44**	**1.96**	**<0.01**	**2.21**	**1.85**	**2.64**	**<0.01**
Non‐Hispanic versus Hispanic ethnicity	1.08	0.91	1.29	0.33	0.99	0.81	1.22	0.94
**Man** versus **Woman**	**2.02**	**1.54**	**2.65**	**<0.01**	**1.51**	**1.05**	**2.19**	**0.02**
Transgender/gender non‐conforming versus cisgender woman	1.17	0.85	1.61	0.32	1.37	0.96	1.93	0.08
White versus Black race	1.04	0.83	1.31	0.68	0.95	0.73	1.24	0.72
**Uninsured** versus **insured status**	**1.2**	**1.04**	**1.37**	**0.01**	1.12	0.96	1.32	0.13

*Note*: Bold values indicate results with *p*‐lvalue <0.05.

^a^ PrEP persistence is defined as having completed at least one clinic visit that resulted in the writing of a PrEP prescription within 24 weeks of the prior visit.

^b^ Persistence in care is defined as having attended at least one clinic visit for any reason within 24 weeks of the prior visit, regardless of whether PrEP was addressed.

Acknowledging variations in the availability of services from all locations, we conducted sensitivity analyses evaluating PrEP persistence after September 2020 (when all clinics had reopened following COVID‐10‐related shutdowns).

All analyses were conducted in R (R Foundation for Statistical Computing, Vienna, Austria).

## 3 RESULTS

### 3.1 Demographics

In Table 1, client characteristics are detailed, highlighting the statistically differences between those seen in the mobile clinics and those seen in the fixed clinic in terms of services initiated, demographics (age, gender, race, ethnicity and country of nativity), baseline HIV, median number of sexual partners and MSM identity. Across all services, the median age was 37.0 years, with 70.7% cisgender men and 54.5% MSM; 24.2% cisgender women. Over half identified as White (57.3%), and 25.6% as Black. Regardless of race, 62.5% identified as Hispanic/Latino, and 54.4% were born outside the United States; 2.1% reported ever having experienced homelessness.

Clients seeking PrEP services were more likely to be younger, cisgender men (86.1%), White (68.3%), Hispanic/Latino (74.9%) and MSM (82.5%) compared to those seeking other services (*p*<0.001). PrEP clients were more likely to be born outside the United States (60.1% vs. 30.5%).

Overall, 90.7% of clients were tested for HIV initially, with a 2.5% rate of new diagnosis. The rate of initial rapid test HIV reactivity was similar for PrEP and other services (2.7% vs. 2.4%; *p* = 0.51). Acute bacterial STIs were present in 19.1% of clients (Chlamydia: 11.3%; Gonorrhoea: 10.4%, Early Syphilis: 3.1%) and higher among PrEP seekers (25.2% vs. 11.3%; *p*<0.01). The median reported number of sexual partners in the last 12 months were 3 overall, and higher among PrEP clients (5 sexual partners vs. 2 sexual partners; *p* = 0.05). Injection drug use was reported by 1.2% overall, with no significant difference between PrEP‐seeking and other clients (1.6% vs. 0.6%; *p* = 0.25). PrEP clients more frequently reported other drug use (Table 1).

### 3.2 Mobile versus fixed clinic

Initial mobile clinic clients differed from fixed clinic clients in demographics (Table 1). Variability existed across mobile sites, reflecting distinct neighbourhood demographics (Table 2).

Reported sex partners, baseline HIV reactivity and STI baseline positivity rates were lower among mobile clinic clients compared with the fixed clinic, with variability by site (Table 1).

### 3.3 Miami versus clinic demographics

To evaluate equitable reach of the fixed and mobile clinic programmes, client demographics were compared to newly diagnosed PLWH in Miami in 2021 and the entire Miami 2022 census population. Our client population did not significantly differ from newly diagnosed PLWH in age, race, ethnicity and US birth. A higher proportion of women was seen in our clinic system compared with the overall group of newly diagnosed PLWH in Miami‐Dade County (24.2% vs. 14.7%). Fixed and mobile clinics had significantly different demographics (Table 1), with the mobile clinic contributing to overall reach, especially to people identifying as Black and cisgender women. The client set was also compared with 2022 overall Miami resident demographics (Table 3). Our client set had a higher proportion of men and people identifying as Black compared with the overall demographics of Miami‐Dade County.

### 3.4 Persistence in care

PrEP clients’ persistence to 24 and 48 weeks was evaluated (Figure 2). The median time in PrEP care was longer for mobile initiators than fixed clinic initiators (72.0 vs. 39.9 weeks, *p*<0.0001). Persistence to 24 weeks was 52.6% for mobile initiators versus 47.3% for fixed clinic. Considering all care (not only appointments for PrEP), persistence to 24 weeks was 73.1% for mobile clinic and 62.6% for fixed clinic (Figure 3). Persistence to 24 and 48 weeks did not significantly differ by race, ethnicity or insurance status, but those with female sex assigned at birth showed lower persistence. Differences in persistence at the mobile compared with fixed site were consistently observed across all comparisons (Figure 4).

In the subgroup analysis of 919 clients who initiated PrEP before March 2022, overall persistence on PrEP was 56.7% at 24 weeks and 41.5% at 48 weeks. Uninsured clients, men and mobile clinic initiators were more likely to continue PrEP to 48 weeks (HR: 1.20, *p* = 0.01; HR: 2.02, *p*<0.01; HR: 1.68, *p*<0.01, respectively). Among the subsample, overall persistence in care (not only appointments for PrEP) was 76.2% to 24 weeks and 55.7% at 48 weeks. Men and mobile clinic initiators had increased continuation (HR: 1.51, *p* = 0.02; HR: 2.21, *p*<0.01). Notably, race and ethnicity were not associated with persistence to 24 or 48 weeks in our analysis.

### 3.5 Pharmacy prescription fill rate

Pharmacy fill data were available for 393 PrEP initiators who filled their prescriptions within four large retail pharmacy chains between March 2020 and March 2023. Dispensation of the initial supply of PrEP medication was confirmed for 356/393 of these clients (90.6%). There were no statistically significant differences in age, gender, race, ethnicity, country of birth, history of acute bacterial STIs, sexual identity or number of reported sexual partners among this subgroup when compared to PrEP initiators overall.

### 3.6 Sensitivity analysis

Analyses including only those clients initiating PrEP after September 2020 (after reopening of all clinic sites following COVID‐19 closures) confirmed the finding of greater persistence to 48 weeks among those initiating PrEP at a mobile PrEP service site (Figure S1).

## 4 DISCUSSION

Our experience with sustained delivery of PrEP services using a mobile clinic model demonstrates that this strategy can extend the equitable reach of PrEP and sexual health services to priority populations not adequately represented in a fixed clinic including Black clients and cisgender women. Persistence was also improved among clients of the mobile clinic and did not differ by race or insurance status. Additionally, as one measure of equitable reach to populations highly impacted by HIV, our combined fixed and mobile cohorts approximated the 2021 demographics of newly diagnosed PLWH in Miami‐Dade County in age, race and ethnicity, and reached proportionally more women (24.2% of clients vs. 13.1% of Miami‐Dade County newly diagnosed PLWH). To our knowledge, this is the first description of client outcomes from an established mobile PrEP clinic programme in the United States.

Mobile clinic strategies have previously been widely deployed for HIV testing and treatment, primary care, and other specialized health services with various models of service delivery worldwide [15, 16, 17, 18, 19, 20, 21, 22, 23, 24]. While mobile clinics have been proposed as a potential strategy for expanding PrEP implementation in the US South [31], data regarding successful models for mobile strategies for PrEP are sparse. A mobile clinic strategy for the delivery of PrEP and sexual health services to adolescent girls and young women in South Africa as part of a differentiated care package has been described in detail, and qualitative data on a venue‐based PrEP mobile strategy focused on MSM has been described in the United States [32], both finding acceptability and feasibility of the strategy. Our mobile clinic strategy is unusual in its urban focus and differs from other proposed models which encourage spur‐of‐the‐moment engagements at events or venues followed by referral for PrEP continuation; our model of regular days and hours at each community site aims to facilitate the development of trust and continuity particularly in areas that have historical experience with and distrust of one‐time or transient community initiatives [28].

Our data show that each of our five mobile clinic locations, selected through a review of local epidemiological data with the Florida Department of Health and community partners, provided unique reach to key populations, reinforcing the importance of strategic neighbourhood positioning. Although all services are offered at each location, services accessed differed significantly by location and by key population. For example, we note that cisgender women, while reached more effectively through the mobile clinic, frequently accessed HIV and STI services independent of PrEP. We consider this non‐PrEP service engagement to be an important opportunity to continue to offer PrEP and eventual potential progression through the “Motivational PrEP Cascade” [33] over time. Future work will evaluate interventions to facilitate the successful transition to PrEP care after initial mobile clinic engagement for other services.

We noted increased persistence to 48 weeks on PrEP and in all services for our mobile clinic cohort, suggesting the added value of the mobile clinic neighbourhood positioning in addition to the other strategies deployed across fixed and mobile sites. Importantly, race and ethnicity were not associated with persistence in our cohorts, and individuals without commercial insurance had similar or better persistence in care compared with those who were insured, suggesting that our navigation and support strategies are successful in overcoming multilevel barriers associated with inequities in PrEP persistence based on race and insurance status reported in other studies [34, 35, 36]. PrEP persistence among women in both the fixed and mobile cohorts was, however, decreased compared with men, consistent with findings from US national pharmacy data [36]. Our measured persistence is, overall, similar or greater than that described for other PrEP populations in fixed clinics in the US South. For example, Burns et al. reported 55% persistence at 3 months, and 37% persistence at 8–12 months from a North Carolina clinic [34], and Rolle et al. reported 32% retention to 6 months for PrEP clients from a municipal clinic in Atlanta [37]. From the US PrEP Demonstration Project, which provided on‐site medication, aggressive follow‐up and incentive payments, persistence to 48 weeks was 43.6% for the Miami site [38]. Notably, our fixed and mobile clinic 24‐week persistence increased to 73.1% and 62.6%, respectively, when all services, not only PrEP continuation, were included in the analysis. Thus, clients who discontinue PrEP temporarily or permanently may continue to receive other HIV and STI prevention care through our clinics.

### 4.1 Limitations

These study findings are subject to several limitations. Defining meaningful measures for real‐world persistence in PrEP care can be difficult as individuals may reasonably elect to discontinue PrEP due to changes in risk behaviour, risk perception or the use of other prevention strategies. Clients may also relocate, change PrEP providers or temporarily stop and then reengage in care, resulting in an underestimate of persistence. Additionally, while clients on oral PrEP are scheduled for quarterly follow‐up visits per US Centers for Disease Control and Prevention guidance, strict cut‐offs based on these recommended visits underestimate real‐world retention in which visits may not exactly coincide with 3‐month intervals, and in which starting and stopping PrEP may be frequent [39].

Fixed and mobile clinic services did not begin simultaneously, and mobile services were suspended due to the COVID‐19 pandemic from March to September 2020. For this reason, we excluded PrEP initiators during this period from Kaplan−Meier analysis. Follow‐up visits were not excluded. Additional analyses including only those initiating PrEP after September 2020 also supported the finding of greater persistence among those initiating PrEP at a mobile PrEP service site.

While this is an observational study and cannot control for variables driving decisions regarding where to seek services, the team providing care, services, protocols and available resources for support are identical across the fixed and mobile clinics. Additionally, while our mobile clinic clients frequently have additional barriers to care that limit their ability to access the fixed site, they may also have positive motivational, personal or social characteristics that are not measured in our data.

## 5 CONCLUSIONS

Through our community‐based mobile clinic model, we achieved sustainable delivery of PrEP care and increased reach to key populations for HIV prevention despite challenges related to insurance, immigration, frequent relocation and other common barriers to medical care. Mobile clinics, combined with other barrier‐lowering approaches, may represent a useful strategy for other jurisdictions facing continued high rates of HIV transmission and with difficulties achieving equitable reach of biomedical prevention. Additional work to understand and overcome contributors to PrEP discontinuation in the mobile clinic context is needed.

## COMPETING INTERESTS

SD‐L declares the following potentially competing interests: Research funding to institution from Gilead Sciences and Merck (for work not related to that presented in this manuscript). All other authors: no competing interests.

## AUTHORS’ CONTRIBUTIONS

SD‐L: conceptualization, formal analysis, funding acquisition, investigation, methodology, project administration, supervision, validation, writing draft and editing. AJ: formal analysis, methodology, supervision, validation, writing draft and editing. KK: conceptualization, project administration. KK: conceptualization, project administration. GN: conceptualization, project administration. SB: conceptualization, project administration. PW: conceptualization, resources, funding acquisition. EK: conceptualization, resources, funding acquisition. MS: conceptualization, resources, funding acquisition, editing.

## FUNDING

The study was supported by the Miami Center for AIDS Research‐P30AI073961 admin supplement to SD‐L.

## Supporting information



Supporting Information

## Data Availability

The data that support the findings of this study are available on request from the corresponding author. The data are not publicly available due to privacy or ethical restrictions.
