# Supplementary material for: An observational cohort study evaluating PrEP reach, engagement and persistence through a community‐based mobile clinic in Miami‐Dade County, Florida
Source: J Int AIDS Soc. 2024 Oct 14;27(10):e26362. doi: 10.1002/jia2.26362 (PMC11473651; doi:10.1002/jia2.26362)
Supplement: Supplementary file 1 — Supporting Information [file JIA2-27-e26362-s001.docx]

Supplemental Figure 1: Persistence in care using follow-up visits only from clients initiating PrEP after September, 2020 (sensitivity analysis)


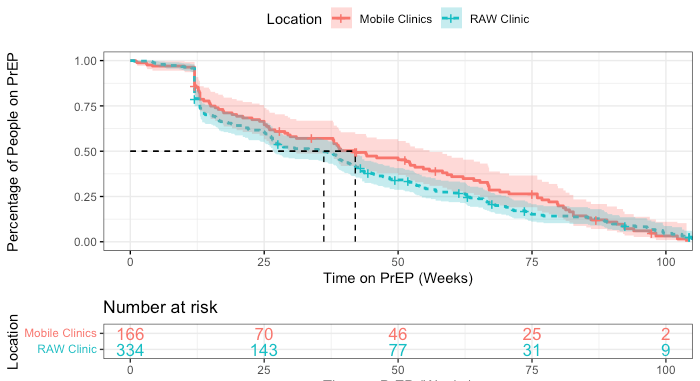


Location

Time in Care (Weeks)

Probability of PrEP Persistence

Mobile Clinics Fixed Clinic

Number at risk

p < 0.0001
